# Filtering large-scale event collections using a combination of supervised and unsupervised learning for event trigger classification

**DOI:** 10.1186/s13326-016-0070-4

**Published:** 2016-05-11

**Authors:** Farrokh Mehryary, Suwisa Kaewphan, Kai Hakala, Filip Ginter

**Affiliations:** Department of Information Technology, University of Turku, Turku, Finland; The University of Turku Graduate School (UTUGS), University of Turku, Turku, Finland; Turku Centre for Computer Science (TUCS), Turku, Finland

**Keywords:** BioNLP, Event extraction, Trigger detection, Word embeddings

## Abstract

**Background:**

Biomedical event extraction is one of the key tasks in biomedical text mining, supporting various applications such as database curation and hypothesis generation. Several systems, some of which have been applied at a large scale, have been introduced to solve this task.

Past studies have shown that the identification of the phrases describing biological processes, also known as trigger detection, is a crucial part of event extraction, and notable overall performance gains can be obtained by solely focusing on this sub-task. In this paper we propose a novel approach for filtering falsely identified triggers from large-scale event databases, thus improving the quality of knowledge extraction.

**Methods:**

Our method relies on state-of-the-art word embeddings, event statistics gathered from the whole biomedical literature, and both supervised and unsupervised machine learning techniques. We focus on EVEX, an event database covering the whole PubMed and PubMed Central Open Access literature containing more than 40 million extracted events. The top most frequent EVEX trigger words are hierarchically clustered, and the resulting cluster tree is pruned to identify words that can never act as triggers regardless of their context. For rarely occurring trigger words we introduce a supervised approach trained on the combination of trigger word classification produced by the unsupervised clustering method and manual annotation.

**Results:**

The method is evaluated on the official test set of BioNLP Shared Task on Event Extraction. The evaluation shows that the method can be used to improve the performance of the state-of-the-art event extraction systems. This successful effort also translates into removing 1,338,075 of potentially incorrect events from EVEX, thus greatly improving the quality of the data. The method is not solely bound to the EVEX resource and can be thus used to improve the quality of any event extraction system or database.

**Availability:**

The data and source code for this work are available at: http://bionlp-www.utu.fi/trigger-clustering/.

**Electronic supplementary material:**

The online version of this article (doi:10.1186/s13326-016-0070-4) contains supplementary material, which is available to authorized users.

## Background

The overwhelming amount of biomedical literature published annually makes it difficult for life science researchers to acquire and maintain a broad view of the field, crossing the boundaries of organism-centered research communities and gathering all of the information that would be relevant for their research. Modern natural language processing (NLP) techniques strive to assist the researchers with scanning the available literature and aggregating the information found within, automatically normalizing the variability of natural language statements. The applications of NLP in life sciences range from automated database curation and content visualization to hypothesis generation and offer intriguing challenges for both NLP and life science communities [1–3].

As a response to the need for advanced literature mining techniques for the biomedical domain, the BioNLP (Biomedical Natural Language Processing) community of researches has emerged. The primary focus of the majority of research within the BioNLP community is to improve information retrieval (IR) and information extraction (IE) in the domain.

In this paper we focus on the task of *event extraction*, a task that has received much attention in BioNLP recently. Event extraction constitutes the identification of biological processes and interactions described in biomedical literature, and their representation as a set of recursive event structures. In its original form, introduced in the 2009 BioNLP Shared Task on Event Extraction (ST) [4], the task focused on gene and protein interactions, such as RNA transcription, regulatory control and post-translational modifications. In subsequent Shared Tasks, while the overall setting remained unchanged, the task has been broadened to cover a large number of additional biological domains and event types [5, 6].

**Fig. 1:**
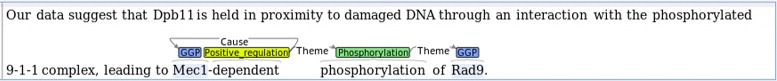
Visualization of a specific event occurrence. Genes and gene products (‘GGPs’) are marked, as well as the trigger words that refer to specific event types. Finally, arrows denote the roles of each argument in the event (e.g. Theme or Cause). (Adapted from [23])

More specifically, event extraction involves detecting mentions of the relevant named entities which are typically genes and gene products (GGPs), the type of their interaction from a small vocabulary of possible types, the *trigger* expression in the text which states the event, and the roles of the participants in the event, e.g. *regulator* or *regulatee*. One of the distinguishing features of events is that they can recursively act as participants of other events, forming recursive tree structures which precisely encode the factual statements in the text, but are a challenging extraction target. An example of an event is shown in Fig. 1.

A number of event extraction systems have been introduced as the result of the series of BioNLP Shared Tasks. Most of these systems focus solely on the immediate textual context of the event candidates, but recently approaches benefiting from bibliome-wide data, either through self-training or post-processing steps, have been introduced as well [7–9]. Unfortunately the recent advancements in this field have been modest, reflecting the complexity of the task. As an example, the best performing system in ST’09, TEES (Turku Event Extraction System) [10], has remained a state-of-the-art approach, winning also several categories in the later Shared Tasks, although the performance of the system has not increased substantially during these years.

Several event extraction systems have been applied at a large scale, extracting millions of events from massive text corpora [11, 12]. These large corpora, typically the totality of PubMed abstracts and PubMed Central full-text articles, contain a number of documents which are partly or entirely out-of-domain for these systems, being unlike the carefully selected data from narrow biological domains on which the systems have been trained. Facing documents from such previously unseen domains, the systems often produce suboptimal output, making what seems to a human like trivial mistakes. Tuning the performance of these systems in the general domain requires further effort.

Here we focus on EVEX [11], an event database covering the whole PubMed and PubMed Central Open Access (PMC-OA) literature, produced using the aforementioned TEES system. Already a casual inspection of EVEX reveals occasional occurrences of obviously incorrect events especially in out-of-domain documents. Previously, Van Landeghem et al. [13] have studied the output of the event extraction systems on general domain data in further detail. Their analysis resulted in a set of rules that can be used to remove or correct erroneous events. Although applying this method produced only an increase of 0.02pp in F-score when evaluated on the official Shared Task data, the consequences on large-scale resources such as EVEX are significant: hundreds of thousands of false events can be excluded, thus greatly improving the quality of the extracted data. This is because the official Shared Task test data does not contain the out-of-domain documents found in the corpora used to build EVEX and many of the error types made by the system will not be seen in the test set output.

Van Landeghem et al. point out that a large portion of the false event predictions originate from the trigger detection phase, i.e. false positive identification of the textual spans expressing the biological processes underlying the events. These, in turn, lead to the generation of false positive events by the system. Here it is important to take into consideration that the top-ranking event extraction systems are based on machine learning and do not uniquely rely on a list of possible “safe” trigger words, which would result in an excessively low recall. Instead, any word can become a trigger word, which occasionally leads to wildly incorrect predictions. These, in turn, are easily spotted by the users of the event databases and decrease the perceived credibility of the resources.

In this paper, we thus focus specifically on the event triggers in the EVEX event database, with the objective of automatically identifying and removing those that are obviously incorrect. To solve this problem, we introduce a novel approach based on word embeddings, bibliome-wide statistics and both supervised and unsupervised machine learning techniques.

Since our method relies on bibliome-wide statistics that should be gathered from a large-scale biomedical event database, it serves as a post-processing step in event extraction pipeline to filter out incorrect events from that database, after the events are extracted.

## Method

In this section, we first introduce the data that is used in this study and then propose a 6-step method to achieve the aforementioned objectives.

In the first five steps of our method, we focus on the top most frequent trigger words which account for 97.1 % of all events in EVEX. In steps 1, 2, 3 and 4 we perform hierarchical clustering of these trigger words and build, analyze and prune the resulting binary tree to categorize these triggers as correct/incorrect. In step 5, we refine these two sets using manual annotation. Finally in step 6, we build a predictive model based on support vector machines (SVM) to classify the triggers as correct or incorrect.

### Data

This study is based on the EVEX resource [11] containing 40,190,858 events of 24 different types such as *binding, positive-regulation, negative-regulation, and phosphorylation*. These events are extracted using the TEES system [14] from 6,392,824 PubMed abstracts and 383,808 PMC-OA full-text articles that were published up to 2012 and which contain at least one gene/gene-product mention. The EVEX resource can be downloaded and browsed online at www.evexdb.org.

**Fig. 2:**
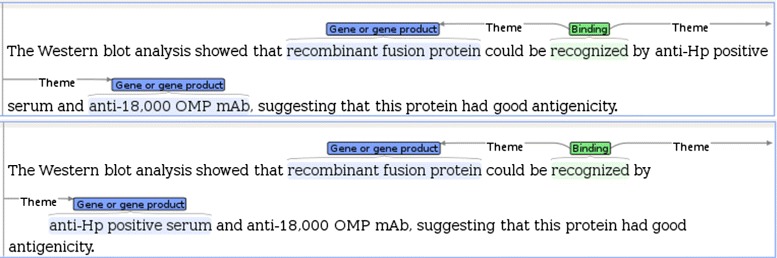
Example sentence with multiple events sharing a single trigger. Two event occurrences extracted from the same trigger word *recognized*

Trained on the ST-data sets, TEES extracts events based on the recognition of an occurrence of a trigger word in the underlying sentence. An event is thus representing the link between the event trigger word and participating argument GGPs. However, one textual span can act as a trigger for multiple events with varying arguments as illustrated in Fig. 2.

In addition, a single unique trigger word, such as *modify*, may have a number of occurrences in the data, acting as a trigger for many events. It is important to note that these events may be of different types. For instance the trigger word *expression* acts as a trigger for both *gene-expression* and *transcription* events, depending on the context.

Throughout this paper, we refer to the total number of extracted events from a trigger as “trigger *frequency*” and to the actual occurrence count of the trigger in the corpus as “trigger *occurrence count*”. Clearly, trigger *frequency* is greater or equal to trigger *occurrence count* since one trigger occurrence can be associated with multiple events. For example, the *frequency* of *expression* is 3,909,759, while its *occurrence count* is 2,736,782. It should be highlighted that the aim of this study is to increase the precision of extracted events, thus the focus is on the trigger *frequency*, i.e. the number of incorrect events that are finally removed from EVEX, when a particular trigger is identified as incorrect.

In total, there are 137,146 unique event triggers (excluding obviously incorrect trigger words that are purely numbers and those which contain unicode special characters). Different trigger words have different *frequency* in the system ranging from 1 to 3,909,759.

As expected, the vast majority of events in EVEX correspond to a small number of highly frequent trigger words, as shown in Table 1. For example, there are only 3,391 trigger words with *frequency* above 300 (i.e. corresponding to at least 300 event occurrences), but these words account for 97.1 % of all events in EVEX. Consequently, when the aim is to increase the precision of the events in EVEX by recognizing *incorrect* trigger words and eliminating them, the focus should be centered on highly frequent trigger words instead of the rare ones. Accordingly, we decided to concentrate on these 3,391 top most frequent trigger words. Limiting ourselves to the top most frequent trigger words allows manual inspection of the hierarchical clustering tree discussed in the following sections.

**Table 1:** Distribution of triggers and their associated event percentages in the EVEX database

Trigger word frequency	EVEX events coverage	Number of trigger
(at least)	percentage	words
100	98.4	6339
200	97.6	4263
300	97.1	3391
400	96.6	2880
500	96.3	2538

Among the trigger words, we will target those which are obviously incorrect, regardless of their context. These could be for example, gene/protein/chemical names, author names or any other words such as *hospital*, *university*, *research*, *diagram*, *box*, *clarify*, *investigate*, *visualization*, *knowledge*, *one* or *please*. The main objective of this study is thus to develop a method that can categorize the trigger words so as to eliminate the obviously incorrect trigger words, thus increasing the precision of the event extraction systems without impacting their recall.

Another interesting aspect when studying the trigger words is to build a general overview of all of the trigger words according to the 24 different event types and to study whether there exist groups/sub-groups of related trigger words which would allow us to define subtypes of the 24 event types. Of specific interest will be studying the groups/sub-groups before and after eliminating incorrect trigger words.

### Hierarchical clustering of top most frequent trigger words

In the first step, we induce a vector space representation for the trigger words, and hierarchically cluster the triggers based on this representation. *Cosine similarity* is used as the clustering metric with the *Ward’s variance minimization algorithm* defining the distances between newly formed clusters. To build the vector space representations, we use the *word2vec* method of distributional semantics introduced by Mikolov et al. [15] and previously applied in the biomedical domain by Pyysalo et al. [16]. The *word2vec* method comprises a simplified neural network model with a linear projection layer and a hierarchical soft-max output prediction layer. The input layer has the width of the vocabulary, while the projection layer has the desired dimensionality of the vector space representation. Upon training, the weight matrix between the input and the projection layer constitutes the word vector space embeddings. The network can be trained in several different regimes, but in this work we use the skip-gram architecture, whereby the network is trained to predict nearby context words, given a single focus word at the center of a sliding window context.

We train the *word2vec* model on the lower-cased texts from the EVEX resource, i.e. all abstracts and full-text articles in which at least one GGP was identified. All *GGP* mentions in the texts are replaced with the *“ggp”* placeholder and all numbers with the *“num”* placeholder to densify the text.

An initial experiment in hierarchical clustering of the top 100 most frequent trigger words revealed that on one hand many coarse/fine grained sub-clusters were formed in a way that each sub-cluster contained trigger words with biologically similar meaning. Many sub-clusters could be clearly associated with a unique event type. On the other hand, many trigger words were clustered together incorrectly, especially for the common *positive-regulation* and *negative-regulation* types (e.g. *increase* and *decrease*) because they have a high similarity in the vector space representation.

To address this issue, we add trigger/event type association information as additional dimensions to the word vectors, thereby affecting the clustering to more closely conform to the event types. To obtain reliable event type distribution for the trigger words, we use the BioNLP Shared Task 2011 (ST’11) *training* and *development* sets [5]. Out of the 1,447 unique trigger words in this data, 995 are single-token trigger words and of these, 828 are actually among the top 3,391 most frequent EVEX trigger words. For these 828 triggers, we append a normalized event type distribution vector to their *word2vec*-based vectors (the vectors for the remaining 2,563 triggers for which a reliable event type information could not be obtained are simply padded with 24 zeroes). Re-clustering with the modified vectors, we notice that *positive-regulation* and *negative-regulation* trigger words are no longer clustered together, obtaining more meaningful clusters with regard to the task at hand.

### Event type vectors of sub-clusters

In this step, event type distribution vectors for all nodes of the binary cluster tree are calculated. For each leaf of the tree (i.e., a trigger word), its corresponding trigger/event type vector is calculated based on the occurrence counts of its respective events in EVEX, and for each intermediate node of the tree (i.e., a sub-cluster), its respective event type vector is calculated by adding trigger/event type vectors of all triggers that belong to it.

Using this information, it is possible to inspect how the tree is organized and whether and how its different branches represent different event types. For example, by checking which element in a sub-cluster’s event type vector has the maximum value, we can tell what is the event type that this sub-cluster is mostly associated with and the level of purity of that cluster. For example, while one sub-cluster can be 98 % *binding* and is thus to a large extent *pure*, another cluster can be 43 % *gene_expression* and cannot be assigned a single predominant type.

### Identifying *possibly incorrect* trigger words

Focusing on 3,391 top most frequent trigger words, in this step we prepare a list of *safe* or *supposedly correct* trigger words and regard the remaining triggers as *possibly incorrect*. This is necessary for pruning the tree and finding the list of *incorrect* trigger words in the next step.

As stated in Section Hierarchical clustering of top most frequent trigger words, by analyzing the ST’11 training and development sets, we obtain a list of 995 unique single-token trigger words. Some of these triggers are overlapping with EVEX triggers. However, our list contains many other trigger words that can not be directly found in the ST’11 sets, but variations of them or variations of their parts can. For instance, *processing* and *co-regulation* are in the EVEX-based list, while *processed* and *regulation* are in the ST’11 sets.

We therefore process BioNLP ST’09 [4], ST’11 [5], and ST’13 [6], *training* and *development* sets, to obtain a set of all single-token ST-trigger words. This trigger set, hereafter *ST-set*, contains 1,092 trigger words. Then we perform the following preprocessing steps on every trigger word in both EVEX and *ST-set*.

1. Remove any punctuation or special characters from the beginning of the trigger word, retaining the rest of the word as the trigger word. For example, *-stimulated* is transformed into *stimulated*.
2. We split each trigger word based on occurrences of the following characters: {‘-’, ‘.’, ‘_’, ‘/’}. For example, *co-express* is divided into *co* and *express*, and similarly *cross-reacts* is divided into *cross* and *reacts*.
3. For every trigger word, each of its split parts is saved if *all* of the following conditions are met:
  1. The part is longer than one character.
  2. The part is not a number.
  3. The part is not in the following stop list: {*32p*, *auto*, *beta*, *cis*, *co*, *cross*, *de*, *double*, *down*, *mono*, *non*, *out*, *poly*, *post*, *re*, *self*, *trans*, *under*}. We obtained this list experimentally by careful examination of the *ST-set*.
4. Finally, we lemmatize all the trigger words, and all of their parts, using the *BioLemmatizer* tool [17] which is specifically developed for the biomedical domain, and record all the produced lemmas for each trigger word.

After the preprocessing, 977 EVEX trigger words that can directly be found in the *ST-set* are regarded as *safe*. The rest of the triggers are regarded as *safe* if their exact form, or one of their parts, or one of the lemmas of their parts can be found in the *ST-set*, or *ST-set* words’ parts or part lemmas. Otherwise, the trigger word is regarded as *possibly incorrect*.

Performing the aforementioned approach resulted in identification of 506 trigger words which were added to the list of *safe* triggers, totaling to a list of 1,483 *safe* trigger words. The 1,908 remaining triggers are regarded as *possibly incorrect*. Table 2 shows some example words from EVEX triggers in our list that are matched against *ST-set* trigger words, parts, or lemmas.

**Table 2:** Examples of matching EVEX trigger words against Shared Task *exact trigger words* or their corresponding *parts/lemmas*

EVEX trigger word	ST’11-trigger word/Part/Lemma
co-transcribed	transcribed
calcium-induced	induced
co-immunoprecipitates	immunoprecipitate
downregulating	downregulate
recognise	recognize
preceding	precede
analyzing	analyse

As discussed earlier, we do not save parts of the EVEX trigger words if they belong to our stop list. The stop list comprises the prefix parts obtained by splitting *ST-set* trigger words, which are *not* themselves *ST-set* trigger words. For example, *cross-link* is a *ST-set* trigger word, but *cross* itself is not a stand-alone *ST-set* trigger, therefore *cross* is included in the stop list. As a contrary example, *up-regulation* is a *ST-set* trigger word, however we did not include *up* in the stop list because *up* itself is a *ST-set* trigger word. We perform such approach because we do not want any EVEX trigger word like *re-X* or *cross-X* (X can be any word) to be matched against *ST-set* words, parts and lemmas, just because it has a *re* or *cross* as a prefix.

### Pruning the tree

Pruning the tree is done using the list of *possibly incorrect* trigger words in four steps:

1. If a trigger word exists in the list of *possibly incorrect* trigger words, its corresponding leaf is marked as *unsafe*, otherwise it is marked as *safe*.
2. If *all* of the children of an intermediate node are marked as *unsafe*, this node (sub-cluster as a whole) is marked as *unsafe* as well, otherwise it is marked as *safe*.
3. All of the descendants of the intermediate nodes that are marked as *unsafe*, are deleted from the tree. The respective trigger words of the deleted leaves are subsequently added to the list of *incorrect* trigger words.
4. After tree pruning, the trigger words of all leaves that remain in the tree, are marked as *safe* and regarded *correct*.

After applying the aforementioned tree pruning algorithm, we obtain a set of *correct* and a set of *incorrect* top most frequent EVEX trigger words.

There is one important aspect in the pruning algorithm. Since the tree is binary, not all of the trigger words that are in the list of *possibly incorrect* triggers were finally regarded as *incorrect* trigger words, because if such a trigger word was clustered near a *safe* trigger word (i.e., had a very small cosine distance to a *safe* trigger word in the feature space), it was not considered as an *incorrect* trigger word and remained in the tree. This helps us to identify more *correct* trigger words.

For example, *co-localization* which is an EVEX trigger word is also a Shared Task trigger word, so it had been marked as *safe* in the matching step, however *colocalization* (another EVEX trigger word) originally had been regarded as *possibly incorrect*, because our matching procedure could not have matched this trigger word (or its lemma) against any *ST-set* trigger word or part or lemma. However, because these two words are extremely similar in the vector space representation, they clustered together in the binary cluster tree. Consequently, since an *unsafe* trigger was clustered with a *safe* trigger, that whole sub-cluster was regarded as *safe* and remained in the tree, so *colocalization* finally is regarded as a *correct* trigger word. To summarize, the tree pruning algorithm causes deletions to be propagated to the upper level nodes of the tree only if *all* of the participating leaves are recognized as *incorrect*.

After pruning, event type vectors for all intermediate nodes of the tree are recalculated so that we can compare the tree before and after pruning.

### Refining *correct* and *incorrect* trigger sets

The output of the tree pruning step are the *correct* and *incorrect* trigger words sets, into which the top most frequent EVEX trigger words are assigned. As discussed in Section Results, our unsupervised method (steps 1-4) increases the precision and F-score of event extraction systems, however it causes a comparatively small drop of recall. This means that some of the *correct* trigger words are erroneously included in the *incorrect* trigger set, thus deleting their corresponding events from EVEX consequently decreases the recall of that event extraction system. As our objective is to increase the precision without decreasing the recall, i.e. we try to avoid removing correct events from EVEX at any cost, we address the issue using manual annotation to refine the results.

#### Manual annotation of triggers

A list of 3,391 trigger words was prepared by extracting the trigger words with *frequency* of at least 300 from EVEX. As discussed in Section Identifying *possibly incorrect* trigger words, 977 of EVEX top most frequent triggers overlap with the *ST-set*. We assume these triggers to be correct and provided the 2,414 remaining trigger words to an annotator with prior experience in biomedical domain annotation.

The annotator performed the manual annotation by deciding for each trigger whether it is *correct* or *incorrect*. On one hand, a trigger is *correct* if its occurrence can lead to the extraction of one or more of the 24 Shared Task event types, i.e. the given trigger word can represent at least one of the ST event types in some context, although in another context they might still be invalid. On the other hand, an *incorrect* trigger cannot express Shared Task events in any context. The annotator was allowed to use any available resources, such as NCBI [18], Gene Ontology [19] and KEGG [20] databases, to support the annotation.

The annotation of the top most frequent EVEX triggers resulted in three categories:

- 2083 triggers were annotated as *correct*.
- 577 triggers were annotated as *incorrect*.
- 731 triggers were not annotated and remained *undecided*.

In a closer look at the annotation data, the most common *correct* triggers are the words specifically used in biomedical domains such as “gene expression”, “regulation” and “transcription” to state the events. The *incorrect* triggers are mostly biomedical entities such as genes, proteins and chemicals. While the majority of the triggers (2660/3391, 78.44 %) can be annotated, the annotator was unable to make a decision for 21.56 % of the triggers. Most of these *undecided* triggers are multiple-meaning words used in both biomedical and generic domains such as “conserved”, “deletion”, and “development”. Thus it is possible to construct hypothetical sentences where these words are valid triggers, but the annotator was not able to find any evidence supporting the use of these words as triggers from the existing literature. While going through all the sentences would be an ideal solution to resolve this issue, it is impossible in practice due to the vast amount of the data.

As this evaluation was conducted by a single annotator, we have not assessed the inter-annotator agreement (IAA) for this task. To our knowledge, the organizers of the BioNLP Shared Task have not reported the IAA for the GE data set either. For the EPI data set (Epigenetics and Post-translational Modifications) the organizers report agreement level of 82 % measured in F-score [21]. This evaluation, however, measures the annotation of the full event structures and no direct conclusions can be made for the trigger annotations.

#### Aggregating unsupervised method results with manual annotation results

In this step, we aggregate the results from tree pruning (Section Pruning the tree) and manual annotation. We naturally prioritize the manual annotation, i.e., in the aggregated data a trigger remains *correct* or *incorrect* if labeled as such in the manual annotation. The *undecided* triggers are assigned using the tree pruning method. As a result, the *final* set is comprised of 2,242 *correct* triggers and 1,149 *incorrect* triggers.

### Classification of low-frequency event triggers

In the previous steps, the focus was on assigning a label for top most frequent trigger words (those with frequency of at least 300) which account for 97.1 % of all EVEX events. However, this demanding manual annotation method can not be applied to the huge number of triggers with lower *frequency* that exist in EVEX. To address this problem, we use support vector machines (SVM) to classify the low-frequency triggers (i.e., triggers with *frequency* below 300). As the training data, we use the aggregated trigger set from the previous section, assigning *correct* and *incorrect* triggers as *positive* and *negative* examples, respectively. Our training set totals 3,391 training examples, consisting of 2,242 positive and 1,149 negative examples. We optimize the model using grid-search combined with cross-validation.

Prior to building the model, we considered two important aspects which should be highlighted here. First, we prefer a conservative predictive model which tends to have a very high positive recall, because if the classifier mispredicts a *correct* trigger as *incorrect*, all of its respective events mistakenly will be deleted from the output of event extraction systems which is very undesirable and that can also have a huge adverse effect on the recall of events. Conversely, if the classifier mistakenly predicts an *incorrect* trigger as *correct*, its respective events will remain in the output of event extraction systems, and in general we prefer to tolerate false events instead of deleting correctly extracted events. Because of this reason, and because our training set is imbalanced, we give weight 10 to the positive class and weight 1 to the negative class during classifier training. These weights are set experimentally during the grid search and classifier optimization. In addition, during optimization, instead of optimizing against F1-score we optimize against F2-score, because F2-score weights recall higher than precision.

Second, from the point of view of event extraction systems, the respective events of the triggers are more important than the trigger words themselves. For instance, misclassifying a correct trigger with *frequency* of 200 will translate into removing 200 correct events, comparing it with the removal of a correct trigger with *frequency* of only 1. Consequently, we consider the precision and recall of respective events (not the triggers) and adjust the parameter optimization and training accordingly:

- During optimization, instead of optimizing against the F2-score of triggers, i.e., calculating F2-score based on the *counts* of true-positives (TP), true-negatives (TN), false-positives (FP) and false-negatives(FN), we optimize against F2-score of trigger *frequency*, i.e., calculating F2-score based on the sum of *frequencies* of TP, TN, FP and FN.
- We give a weight to each training example by calculating the *logarithm of its frequency*.
  Thus the training examples with higher weights, i.e. higher event frequency, will be regarded more important than lower weight examples, those with lower event frequency. In other words, classifier will be penalized more on misclassifying the frequent trigger words than lesser ones during training and k-fold cross-validations. As a result, the classifier is trained towards better performance on more frequent triggers while we intentionally do not give the trigger frequency as a feature to the classifier.

Below is the set of features used by the classifier.

1. *word2vec*-based vector for each trigger, which is exactly the same vector discussed in Section Hierarchical clustering of top most frequent trigger words.
2. If the trigger word (or its lemma or its parts or lemmas of its parts) can be matched against *ST-set* words/parts/lemmas (according to Section Identifying *possibly incorrect* trigger words) this feature is 1, otherwise it is zero.
3. The value for this feature is calculated as following:
  *feature_value = occurrence count of trigger / X*
  where *X* is the sum of occurrences of the word in all PubMed abstracts and PMC-OA full text articles published up to 2013, regardless of being recognized as a trigger word in the underlying sentences or not.
  For many incorrect trigger words, this feature will have a very low value. For example, for an incorrect trigger word like *hospital* which is in EVEX (not in training set), the value will be (928 / 1,266,408) = 0.0007.
4. For this feature, we first extract the set of single-token triggers with length of more than 6 characters in the *ST-set*, introduced in Section Identifying *possibly incorrect* trigger words. Then, for each training example we calculate the feature value as following:
  *feature_value = length(trigger word) / (Y + 1)*
  where *Y* is the minimum *edit distance (Levenshtein distance)* of the trigger word to the words in the previously created set. For all training examples that belong to the *ST-set*, we assume *Y* to be zero.
  The longer the trigger word, and the smaller its minimum edit distance, the higher will be the value of this feature.
  This feature is beneficial for example in the case of a misspelled trigger (e.g., *phosphoryalation* instead of *phosphorylation*), which is not recognized correctly by our matching protocol discussed in Section Identifying *possibly incorrect* trigger words.
5. Number of alphabetic characters divided by the length of the trigger word.

We perform a grid-search combined with 5-fold cross-validation to optimize the classifier and find the best hyper-parameters for the model (*kernel type*, *C value*, and the *gamma parameter* for RBF-kernel) against the F2-score of trigger event frequency. Subsequently, we train the classifier using the best parameter values on all available training examples.

## Results

In this section, we discuss the results in four parts. First, in Section Evaluation of event filtering, we evaluate the impact of trigger pruning on event extraction systems. We then evaluate our predictive model and investigate the effect of event filtering on the EVEX resource in Sections Evaluation of low-frequency trigger classification and Evaluation of event removal on the EVEX resource. Finally, in Section Tree organization before/after pruning we examine the trigger cluster tree organization before and after the pruning.

### Evaluation of event filtering

#### Evaluation method

We evaluate the impact of trigger pruning on event extraction using the official test sets of the BioNLP ST’11 and GENIA Event Extraction (GE) Shared Tasks (ST’13). As the basis we consider the outputs of the *TEES system* entry [10, 14] in 2011 (3rd place) and in 2013 (2nd place) GE tasks and, for the 2013 Shared Task, also the winning *EVEX* entry [7]. We prune the outputs of these systems by removing events whose trigger words are identified as incorrect using the aforementioned algorithm and evaluate the resulting pruned set of events using the official evaluation services of the respective Shared Task on the held-out test sets. The results are shown in Table 3.

**Table 3:** Performance comparison of the different pruning approaches and the baseline methods (TEES/EVEX) on the official BioNLP Shared Task GE data sets

	Predictions	Precision	Recall	F1-score
TEES-2011 (Shared Task 2011)	Original TEES	61.76	48.78	54.51
	Pruned-TEES (Unsupervised Method)	62.39	48.75	54.74
	Pruned-TEES (Manual Annotation Method)	62.04	48.78	54.62
	Pruned-TEES (Aggregation Method)	62.26	48.78	54.70
	Pruned-TEES (Aggregation Method + SVM)	62.27	48.78	54.71
TEES-2013 (Shared Task 2013)	Original TEES	56.32	46.17	50.74
	Pruned-TEES (Unsupervised Method)	57.13	46.02	50.97
	Pruned-TEES (Manual Annotation Method)	56.63	46.17	50.87
	Pruned-TEES (Aggregation Method)	56.97	46.17	51.00
	Pruned-TEES (Aggregation Method + SVM)	57.01	46.17	51.02
EVEX-2013 (Shared Task 2013)	Original EVEX	58.03	45.44	50.97
	Pruned-EVEX (Unsupervised Method)	58.77	45.29	51.15
	Pruned-EVEX (Manual Annotation Method)	58.32	45.44	51.08
	Pruned-EVEX (Aggregation Method)	58.66	45.44	51.21
	Pruned-EVEX (Aggregation Method + SVM)	58.71	45.44	51.23

It should be highlighted that naturally the magnitude of the F-score improvements is modest, as the top-ranking systems are well optimized and major improvements have been difficult to achieve regardless of the approach. Note also that a filtering approach such as the one proposed in this paper cannot increase recall because it is unable to produce new events. Our main focus thus is on *improving the precisionwhile trying to retain the recall*, aiming to increase the credibility of large-scale event extraction systems in general.

#### Evaluation of unsupervised method

In this section, we investigate the effect of removing triggers from event extraction systems using the set of *incorrect* trigger words obtained from our unsupervised method in Section Pruning the tree.

As shown in Table 3, in all three instances (comparing our *unsupervised method* against the *TEES* system’s predictions on tasks 2011 and 2013, and the *EVEX* system’s predictions on task 2013), we see an improvement in both precision and F-score with a relatively small drop in recall. Especially for the ST’13, the pruned *TEES* system (+0.23pp F-score over *TEES*) matches in performance with the winning 2013 *EVEX* system. Since the *EVEX* system was also based on TEES, it is interesting to note that we have matched these improvements using a different approach. Finally, the pruned *EVEX* system (+0.18pp F-score over the *EVEX* entry) establishes a new top score on the task.

#### Evaluation of manual annotation method

In this section we investigate the effects on event extraction if we rely our method solely on the manual annotation results. We remove events from those three aforementioned event extraction system outputs, using the set of trigger words that were annotated as *incorrect* by the human annotator.

As shown in Table 3, in all three instances (comparing *manual annotation method* against the *TEES* system’s predictions on tasks 2011 and 2013, and the *EVEX* system’s predictions on task 2013), manual annotation retains the recall, which is obviously a better result than our unsupervised method. However in all three instances, its precision and F-score is less than the precision and F-score of our unsupervised method.

The preserved recall suggest that our annotation strongly agrees with the ST annotation guidelines. However, the higher precision of the unsupervised pruning strategy shows that some cases not clear for a human annotator, can be classified with this method.

This is exactly what we had anticipated. As precise annotation was not possible for many trigger words, we have 731 *undecided* top most frequent triggers, and many *incorrect* trigger words might actually be among them.

To summarize, the manual annotation has produced an *almost pure but incomplete* set of *incorrect* trigger words. In comparison to original event extraction system performances, our manual annotation method does increase the precision and F-score while retaining the recall, but its precision and F-score are not as high as our unsupervised method.

#### Evaluation of aggregation method

As shown in the previous sections, our unsupervised method increases the precision and F-score, but slightly drops the recall, whereas the manual annotation alone retains recall with lesser increase in precision. In this section, we investigate the effect of event filtering using the set of *incorrect* triggers obtained from the aggregation method discussed in Section Aggregating unsupervised method results with manual annotation results.

As shown in Table 3, in comparison with the *TEES* performance on ST’11 and ST’13, and the *EVEX* performance on ST’13, the aggregation method retains the recall and increases the precision and F-score. Interestingly, in all three cases, in comparison with manual annotation method it has a higher precision and F-score. Consequently, we conclude that our unsupervised method is indeed able to find *incorrect* trigger words elusive to the human annotator.

If we compare the aggregation method performance with our unsupervised method performance, we notice that in all three instances, it does have a higher recall and in two cases also higher F-score. In one case the unsupervised method alone reaches the highest F-score. This might be due to trigger words that we have annotated as *correct*, but are used in wrong event types by the underlying even extraction system, thus resulting in lower precision.

As a conclusion, while all of our methods establish new top scores on 2013 tasks, the aggregation method is the best among them. It retains the recall, increases the precision and has the best F-score in two cases out of three.

### Evaluation of low-frequency trigger classification

As stated in Section Classification of low-frequency event triggers, we use all 3,391 top most EVEX frequent triggers to train the classifier and aim to apply it on those triggers with *frequency* below 300.

Similar to the previous evaluations, we first evaluate the classifier performance against the Shared Task test sets as an end-to-end system together with the aggregation method. For this aim, we apply the trained classifier to predict labels for EVEX triggers with frequency below 300. This results in identification of 16,674 negative (supposed to be incorrect) triggers with total frequency of 232,748 respective events in EVEX. The rest of the triggers were predicted as correct. Then, we prune the output of event extraction systems using these recognized incorrect triggers and incorrect triggers obtained by aggregation method.

Results for this experiment are shown in Table 3. Comparison of this method (*Aggregation Method + SVM* entries in the table) against our aggregation method, the previously best approach, shows slight increase in both precision and F-score in all three cases while retaining the same recall. Thus, the classifier is able to recognize some previously undetected *incorrect* trigger words, giving us the most complete set of *incorrect* trigger words.

As this processing step focuses specifically on low-frequency (rare) triggers, unlikely to be found in the carefully selected Shared Task data sets, the performance improvement is small, as anticipated. However, we expect the outcome to be more significant in large-scale event extraction and to show this we conduct another evaluation based on the EVEX resource.

In the second evaluation we form an evaluation set by randomly selecting 700 words from the triggers with *frequency* less than 300 in EVEX and use the same manual annotation procedure discussed in Section Manual annotation of triggers to divide them into positive (*correct*) and negative (*incorrect*) sets.

The annotation resulted in 363 *correct* and 233 *incorrect* triggers. For 104 triggers our annotator was unable to assign a label. Even though, in terms of our annotation protocol, the triggers are divided into three independent classes, for simplicity we exclude the 104 *undecidable* trigger words from our test set and use only the 596 remaining words.

The performance evaluation results against the test set are shown in three different tables.

- Table 4 shows the counts and respective event frequencies of true-positives, true-negatives, false-positives and false-negatives.

**Table 4:** Trigger/event classification performance, measured on the EVEX test set: The first column (Count) shows prediction results based on the counts of trigger words (test set examples). The second column (Sum of frequency) shows the number of respective events of those triggers in the EVEX database. For instance, the first row (True-Positive) shows that the classifier has correctly predicted 352 test set trigger words to be *correct triggers*, while these words account for 4,602 extracted events in the EVEX resource

	Count	Sum of frequency
		(Number of events)
True-Positive	352	4602
True-Negative	99	679
False-Positive	134	850
False-Negative	11	115
Total	596	6246
- Table 5 shows the performance in terms of classification of triggers. Precision, recall and F2-score in this table are calculated based on the counts of the predicted triggers.

**Table 5:** Trigger classification performance on the EVEX resource based on trigger counts (test set examples). The prediction measures in this table are calculated based on the values in the first column of Table 4. This table shows how well the classifier is able to classify and distinguish between *correct* and *incorrect* trigger words. The last column (Support) shows that there are 363 *correct* and 233 *incorrect* trigger words in the test set, i.e, 596 in total

	Precision	Recall	F2-score	Support
Negative (incorrect)	0.90	0.42	0.48	233
Positive (correct)	0.72	0.97	0.91	363
Weighted averages, total	0.79	0.76	0.74	596
- Table 6 shows the performance in terms of classification of events. Precision, recall and F2-score in this table are calculated based on the event frequencies of the predicted triggers (i.e., based on the sum of frequencies of TP, TN, FP and FN).

**Table 6:** Classification performance on the EVEX resource based on the respective event counts in the EVEX database. This table shows how well the classifier will perform the prediction, preserving *correct* and eliminating *incorrect* respective events from the EVEX database. The prediction measures in this table are calculated based on the values in the second column of Table 4. The last column (Support) shows that there are 1,529 *incorrect* and 4,717 *correct* corresponding events in the EVEX database (6,246 in total) which are extracted based on those 596 trigger words in the test set

	Precision	Recall	F2-score	Support
Negative (incorrect)	0.86	0.44	0.49	1529
Positive (correct)	0.84	0.98	0.95	4717
Weighted averages, total	0.85	0.77	0.77	6246

As mentioned in Section Classification of low-frequency event triggers, from the event extraction point of view, the event frequencies are more important than the unique trigger words themselves. Thus, results listed in Table 6 are the most relevant for examining the performance of the classifier. As this is also the evaluation metric the classifier hyper-parameters were optimized against, the numbers in Table 6 are generally higher than in Table 5.

We can see that the classifier achieves recall of 0.98 for the positive class, i.e. the *correct* triggers as shown in Table 6. This result suggests that we have succeeded in our goal of preserving as much of the true events as possible. Besides, the classifier also reaches recall of 0.44 for the *incorrect* triggers, i.e. we are able to detect and exclude almost half of the events with false triggers in this evaluation set.

### Evaluation of event removal on the EVEX resource

In this section we investigate the impact of removing events from the EVEX resource based on all trigger words recognized as *incorrect*.

Even though our manual annotation or aggregation methods are able to preserve the recall when evaluated against official predictions of Shared Task test sets, it is not guaranteed that the same performance will be achieved when applying them on a large-scale resource such as EVEX. In fact there might be *correct* triggers which are not present in ST’11 or ST’13 test sets, but are mistakenly labeled as *incorrect* by the human annotator, our unsupervised method or the classifier. Consequently, in the evaluation against official Shared Task test sets, we do not delete these triggers and do not detect any drop in recall. However based on our evaluation results, we are optimistic that most of the correct events will be preserved if the method is applied on the EVEX resource.

To investigate the impact of event removal on EVEX, for top most frequent triggers (accounting for 97.1 % of all EVEX events), we rely on our aggregation method which had the best performance. The aggregation method resulted in labeling 1,149 triggers as incorrect and these account for 1,105,327 events in EVEX.

For the rest of EVEX triggers (*low frequency* triggers accounting for 2.9 % of all EVEX events), we use the classifier. However, the classifier could not be applied to 48,960 triggers with 122,344 respective events (0.3 % of all EVEX events). These words have less than 5 occurrences in the corpus used for training the *word2vec* model, and thus do not have a corresponding vector representation, required by the classifier. Applying the classifier on the rest of low frequency triggers (accounting for 2.6 % of all EVEX events) resulted in identification of 16,674 *incorrect* triggers with 232,748 events in EVEX.

Consequently, in total we have been able to identify 17,823 expected to be incorrect triggers in the whole EVEX resource with 1,338,075 events which constitutes 3.3 % of all events in EVEX.

### Tree organization before/after pruning

In this section we address two questions. First, how the resulting binary cluster tree differs before and after the pruning, and second, whether we can define new event subtypes based on the organization of sub-clusters in different branches of the tree. For these aims, we visualize the tree before and after pruning up to the depth of 9 using the *Dendroscope* software [22]. We label every intermediate node of the tree with its *mostly associated event type* and the *level of purity* of that sub-cluster (see Additional file 1 for diagrams of the tree).

As expected, in both trees we notice that trigger words of same event types are clustered together, to some extent. By considering the length of the shortest path in tree as a basic distant measure, we observe that sub-clusters of similar/related event types are closer in the tree, while sub-clusters of different event types are located far. For instance, triggers for expressing different types of post-translational modifications events (e.g., “phosphorylation”, “DNA-methylation”, “glycosylation”, “acetylation”) are clustered together, far from trigger words for expressing “positive/negative regulation” or “binding”. Similarly, sub-clusters of “gene-expression”, “transcription” and “localization” trigger event types are close in the tree. We observe that before pruning the tree, sub-clusters are not pure. For example, many trigger words for “positive regulation” events are often clustered together with the ones for “negative regulation” events. By removing the sub-clusters of purely incorrect triggers, i.e. pruning, the sub-clusters in the middle levels of the tree become purer and are for the most part, associated with the same event type which signifies the possibility of identifying some of the event subtypes.

We thus continue the analysis on the associated events in the sub-tree anticipating to recognize the patterns. However, to our surprise, there is no clear signal in the sub-clusters that would signify any of the subtypes. As a result, we thus do not pursue further analysis on the trees. To conclude, our pruning algorithm yields a meaningful tree which can distinguish different event types into sub-clusters, however, the resulting clusters could not be used to identify event subtypes.

## Conclusions

In this paper, we propose a method which can be used for identification of incorrect trigger words and removing incorrect events from the output of large-scale event extraction systems.

Our unsupervised method achieves a modest improvement over the winning system of the BioNLP 2013 Shared Task on GENIA event extraction and establishes a new top score on the task. The aggregation of manual annotation results with our unsupervised method results, further increases the precision and F-score of the unsupervised method. Besides, the unsupervised method decreases the original recall when evaluated against official predictions of Shared Task test sets, while our aggregation method retains it.

Because the highly demanding manual annotation is not possible for all EVEX trigger words, we build a SVM classifier for predicting *incorrect* triggers among low-frequency EVEX triggers. While having 0.98 positive recall which translates to preserving a huge proportion of correct events, the classifier has 0.44 negative recall, meaning that it is able to identify about half of the *incorrect* events.

Combining the results of our aggregation method with *incorrect* trigger words identified by applying the classifier on all low frequency EVEX triggers, results in recognition of 17,823 expected to be incorrect triggers with 1,338,075 respective events which constitutes about 3.3 % of all events in EVEX resource.

In this paper we have only discussed the identification of the incorrect triggers and the outcome of removing these triggers from a large-scale event resource. Although our evaluation shows only minimal drop in recall, bluntly removing the corresponding events might have unwanted effects. As the EVEX resource ranks the events shown to the users based on a scoring system derived from the TEES classification confidence, we would thus as a future work like to investigate how to incorporate these new findings in the ranking. This would let us, instead of completely abolishing the likely incorrect events, only to decrease their scoring and conserve them for those use cases that demand extremely high recall, but can overcome the noise in the data.

Another direction is to investigate the different event types in more detail. We hope this study will give us a better insight of whether the method can be adapted to also correct mistyped events, thus increasing the precision even further. For instance, it is possible that a detected *regulation* trigger should in fact be classified as *positive-regulation*, a subtype of *regulation*, but the used trigger detector has not been able to make this distinction. By observing how the given trigger word is located in the hierarchical cluster tree, these errors could be possibly corrected.

As the distributional semantics research is progressing towards better representations of phrases and larger text sections in addition to word-level embeddings, it might be possible in the future to instead of judging the trigger words globally, to focus only on certain types of contexts giving us the ability to make more precise decisions.

## Availability of supporting data

The source code and data sets supporting the results of this article are available at the Turku BioNLP group website at: http://bionlp-www.utu.fi/trigger-clustering/.

## Additional file

Additional file 1 This tar file contains images of the binary cluster tree, before and after the pruning. The HowToInterpretTreeDiagrams.txt file describes how the diagrams should be interpreted. (TAR 1290 kb)
