# Supplementary figures and images for: Filtering large-scale event collections using a combination of supervised and unsupervised learning for event trigger classification
Source: J Biomed Semantics. 2016 May 11;7:27. doi: 10.1186/s13326-016-0070-4 (PMC4864999; doi:10.1186/s13326-016-0070-4)

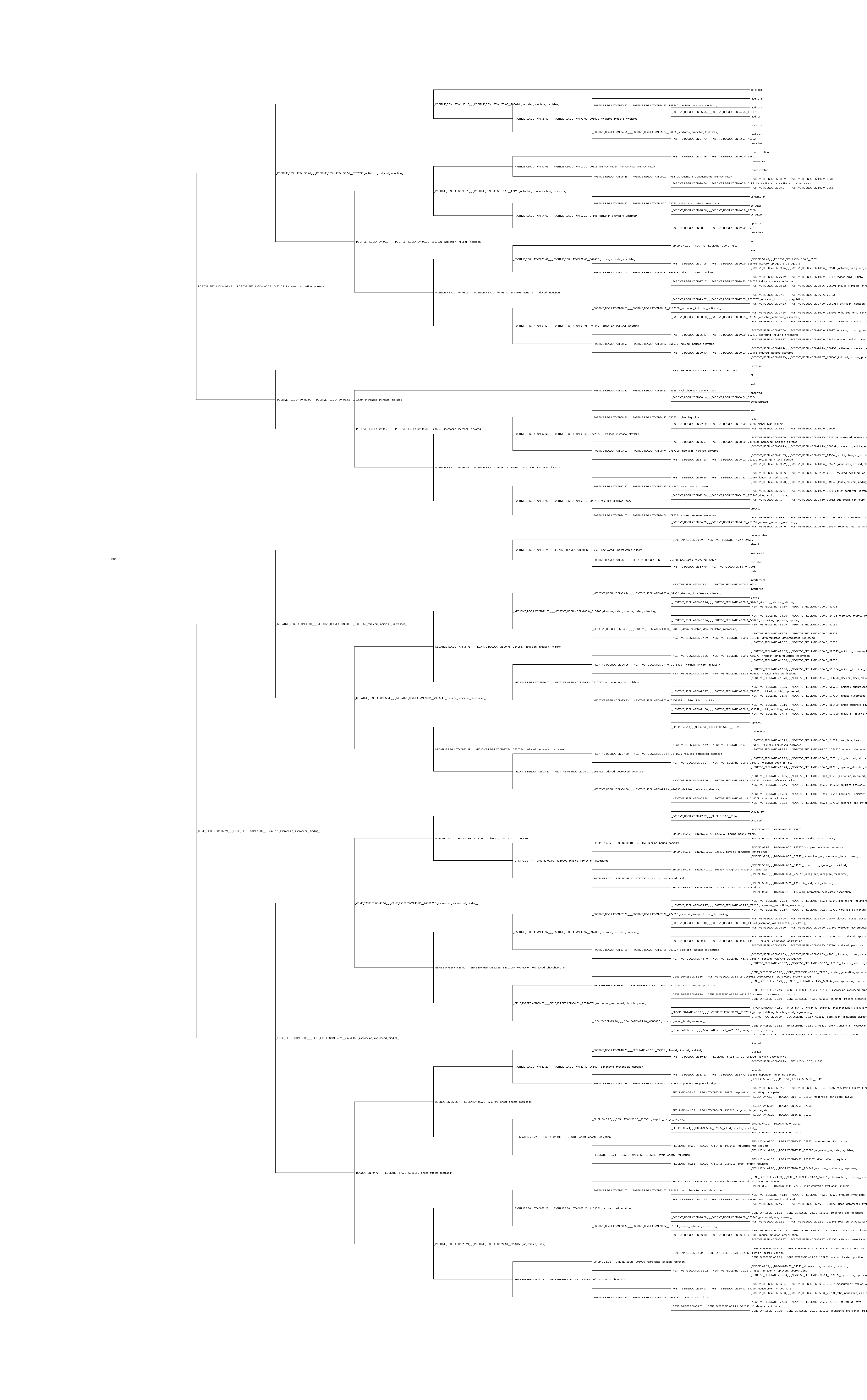

Supplement: Additional file 1 — This tar file contains images of the binary cluster tree, before and after the pruning. The HowToInterpretTreeDiagrams.txt file describes how the diagrams should be interpreted. (TAR 1290 kb) [file 13326_2016_70_MOESM1_ESM.tar › TreeBeforePruning.png]
